# CGRP-targeted therapeutics for migraine: a clinical trial landscape analysis

**DOI:** 10.1186/s12967-025-07123-9

**Published:** 2025-10-15

**Authors:** Jun Li, Liping Huang, Xueyuan Guo, Ruining Wu, Zicheng Liu, Suhang Xie, Gang Wang

**Affiliations:** Department of Rehabilitation Medicine, The First Medical Center, Chinese PLA General Hospital, Beijing, 100853 China

To the editor,

Migraine, a complex neurovascular disorder, affects 14% of the global population—over 1 billion people worldwide [1]. Patients with migraine face stigma, resulting in greater disability, diminished quality of life, elevated depression and anxiety, reduced medical engagement, and suboptimal treatment results [2]. Traditional oral prophylactic medications,including anticonvulsants, antidepressants, calcium channel blockers, and beta-blockers, despite advancements in acute and preventive treatments, a considerable proportion of patients remain refractory to conventional therapies.Calcitonin gene-related peptide (CGRP), a potent vasodilatory neuropeptide, has emerged as a pivotal therapeutic target in migraine pathogenesis. CGRP-targeted therapies (CGRPTT), categorized into monoclonal antibodies (mAbs) and small-molecule CGRP receptor antagonists (gepants), have revolutionized migraine prophylaxis by offering targeted, pathogenesis-based treatment with favorable safety profiles [3]. As the diversity of CGRPTT candidates expands rapidly, a systematic clinical trial landscape analysis is imperative to delineate current research trends and inform future development of CGRPTT.

We systematically searched INFORMA Database, for interventional trials investigating CGRPTT in migraine (last search: Aug 1, 2025). The search strategy included terms such as “Calcitonin gene-related peptide” OR “CGRP” and “migraine”.

A total of 343 eligible interventional clinical trial of CGRPTT for migraine were identified.The landscape reveals several key trends. Temporal trends indicate a surge in Phase Ⅳ trials (e.g., 13 in 2022, 14 in 2020) post-2015, aligning with FDA approvals of erenumab (2018) and subsequent CGRP mAbs/gepants, while early-phase trials peaked earlier (e.g., 8 Phase I in 2008) (Fig. 1A). The US dominated trial sites (201 studies), followed by Italy (65) and Spain (55), suggesting regional disparities in research investment (Fig. 1B). Funding was overwhelmingly industry-driven (222 by top 20 pharma, 58.3%), with minimal government/academic involvement (0.2%, e.g., 1 government-funded trial), emphasizing commercial prioritization over public health-oriented research (Fig. 1C). Geographical distribution shows a predominance of multinational studies, with 256 single-country trials (74.6%, mostly the US) and 30 trials spanning 11–15 countries (8.7%), reflecting global interest yet industry-led concentration in high-resource regions (Fig. 1D). Trial status data show 258 completed studies (75.2%) versus 41 active trials (12.0%), underscoring maturation of this therapeutic class, though 18 terminations (5.2%, e.g., telcagepant due to hepatotoxicity) highlight safety vetting (Fig. 1E). Drug-specific distribution favors erenumab (71 trials, 20.9%), fremanezumab (51 trials, 15.0%), and galcanezumab (43trials, 12.7%), with gepants (e.g., rimegepant: 40 trials, 11.8%) gaining traction (Fig. 1F).

**Fig. 1:**
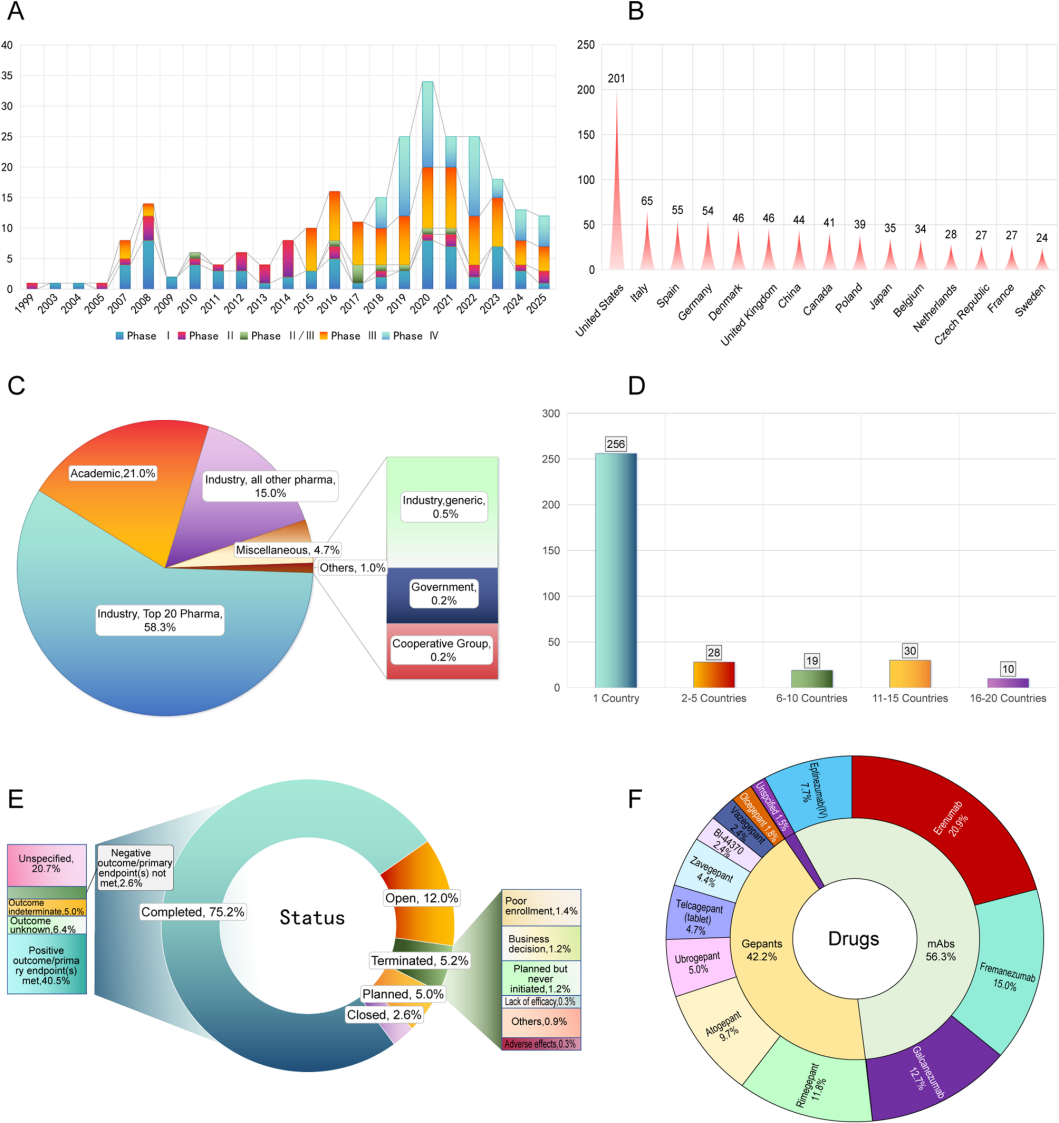
Overview and Outlook for Clinical Trials of CGRPTT for Migraine. **A** Trends in Newly Initiated Clinical Trials by Year and trial phases. **B** Global distribution of CGRPTT for Migraine. **C** Distribution of funding sources, with academic institutions. **D** Conduct of Single-Center/Multi-Center Trials. **E** Distribution of trial status of CGRPTT for Migraine;**F.**Specific types and names of CGRPTT for Migraine

The analyzed trials consistently demonstrate high efficacy for CGRP-targeted therapies in reducing monthly migraine days (MMD) and acute medication use versus placebo, particularly for mAbs in chronic migraine prevention.Although both the European Headache Federation and the American Headache Society recommend CGRP targeted therapies as a first-line migraine prevention, very few countries have incorporated this strategy, mostly due to cost issues and ensuing reimbursement policies, insurance coverage limitations, and substantial patient out-of-pocket expenses [4]. We excluded non-interventional studies and real-world data registries, potentially missing long-term safety or comparative effectiveness insights beyond controlled settings. The geographic analysis reflects trial site locations, not necessarily patient enrolment diversity or regional disease burden.

This analysis confirms CGRP-targeted therapies as a transformative paradigm in migraine management, driven by robust efficacy data and rapid clinical translation. However, significant challenges remain regarding equitable global access, cost-effectiveness, and managing non-responders. Future research must prioritize pragmatic trials in diverse populations, direct comparative effectiveness studies between mAbs and gepants (both preventive and acute), and long-term safety surveillance. Integration with emerging preclinical targets (PACAP, KATP) and leveraging real-world data for personalized treatment algorithms represent crucial next steps towards optimizing migraine care and reducing its global disability burden [5].

## Data Availability

All data used and/or analyzed in this manuscript is publicly available on the Trialtrove database(http://www.pharmaintelligence.informa.com/).

## References

[1] Stovner LJ, Hagen K, Linde M, et al. The global prevalence of headache: an update, with analysis of the influences of methodological factors on prevalence estimates. J Headache Pain. 2022;23(1):34. 10.1186/s10194-022-01402-2 35410119 10.1186/s10194-022-01402-2 PMC9004186

[2] Shapiro RE, Nicholson RA, Seng EK, et al. Migraine-related stigma and its relationship to disability, interictal burden, and quality of life: results of the OVERCOME (US) study. Neurology. 2024;102:e208074. 38232340 10.1212/WNL.0000000000208074 PMC11097761

[3] Tanei T, Fuse Y, Maesawa S, et al. Real-world clinical results of CGRP monoclonal antibody treatment for medication overuse headache of migraine without abrupt drug discontinuation and no hospitalization. 2024;10(22):e40190. 10.1016/j.heliyon.2024.e40190 PMC11693917 39748981

[4] Versijpt J, Paemeleire K, Reuter U, et al. Calcitonin gene-related peptide-targeted therapy in migraine: current role and future perspectives. Lancet 2025;405(10483):1014–1026. 10.1016/S0140-6736(25)00109-6 40121062

[5] Silvestro M, Iannone LF, Orologio I, et al. Migraine treatment: towards new pharmacological targets. Int J Mol Sci. 2023;24(15):12268. 37569648 10.3390/ijms241512268 PMC10418850

